# Comparative survival analysis of preoperative and postoperative radiotherapy in stage II-III rectal cancer on the basis of long-term population data

**DOI:** 10.1038/s41598-018-35493-2

**Published:** 2018-11-21

**Authors:** Yu Jin Lim, Youngkyong Kim, Moonkyoo Kong

**Affiliations:** Department of Radiation Oncology, Kyung Hee University Medical Center, Kyung Hee University School of Medicine, Seoul, Republic of Korea

## Abstract

This study compared long-term population-based survival outcomes of preoperative and postoperative radiotherapy (RT) approaches in rectal cancer. Patients with stage II-III rectal cancer between 1998 and 2013 were identified using the Surveillance, Epidemiology, and End Results database. Overall survival (OS) and disease-specific survival (DSS) rates were estimated in propensity-matched study population according to the use of RT. Among the 28,320 eligible patients, a total of 18,400 patients were identified from propensity score matching process balancing the distribution of prognostic covariates. The 10-year OS and DSS rates were higher in patients with preoperative RT than the postoperative group (51.6% vs. 49.8% with *P* < 0.001, and 65.4% vs. 64.8% with *P* = 0.037, respectively). However, in multivariate analysis, selection of combined RT sequence did not affect the survival (hazard ratio [HR] 1.04 and 95% confidence interval [CI] 0.98−1.10 for OS; HR 0.97 and 95% CI 0.90−1.05 for DSS). Regarding hazard rate functions of cancer-specific mortality, the overall time-course risks after preoperative and postoperative RT were comparable. This study provides additional insight into the long-term prognostic implications of the two RT strategies, suggesting that the sequence of RT does not lead to differential survival in stage II-III rectal cancer.

## Introduction

According to the recent cancer statistics, colorectal cancer is the 3^rd^ most common cancer in the United States^1^. Although incidence and death rates have decreased, colorectal cancer is still a leading cause of cancer deaths^2^. Approximately 39,910 new rectal cancer cases are reported annually^1^, and most are diagnosed as adenocarcinoma. In stage II-III rectal cancer, a multidisciplinary approach including surgical resection, radiotherapy (RT), and chemotherapy is the cornerstone of curative aim^3^. The advent of total mesorectal excision (TME) and combined use of RT or chemoradiotherapy for high-risk patients have improved oncologic outcomes over several decades^4,5^.

Recently, preoperative use of RT has become the preferred option for locally advanced rectal adenocarcinomas^6^. Given that the Dutch trials demonstrated improved local control with neoadjuvant RT^7,8^, three phase III randomized controlled trials, including the German CAO/ARO/AIO-94, National Surgical Adjuvant Breast and Bowel Project (NSABP) R-03, and a Korean study, compared pre- and postoperative use of RT^9–11^. Therapeutic efficacy was evaluated with locoregional recurrence, down-staging, survival, sphincter preservation, and toxicity, but some contradictory results were observed. Moreover, the NSABP R-03 trial was closed early due to an under-recruitment problem, and the number of patients analyzed in the Korean trial was relatively small^10^. Therefore, the recommendation for preoperative treatment is based mainly on the German trial^9^. There was a meta-analysis of the three randomized trials, but interpretation was limited due to the small number of eligible studies^12^.

Survival improvement is one important element in assessing the effectiveness of treatment. However, an updated analysis of the German trial, with a median follow-up duration of 11 years, demonstrated that survival outcomes were not different between preoperative and postoperative strategies^13^. Despite widespread use of the preoperative approach, a recent report from the National Cancer Data Base stated that approximately only one-half of stage II-III rectal cancer patients underwent RT before surgery in the United States^14^. Thus, we used the Surveillance, Epidemiology, and End Results (SEER) program, a nation-wide cancer database from the United States^15^, to evaluate the potential of improved survival with preoperative RT in comparison with postoperative treatment in stage II-III rectal cancer. Since some confounding factors can affect the receipt of preoperative or postoperative options, propensity scores were calculated and adjusted. Baseline hazard rate function plots were used to elucidate time-course changes of cancer-related mortality risks. Our matched comparison analysis of long-term survival outcomes provides additional knowledge for optimizing the sequence of RT combined with surgery in locally advanced rectal cancer.

## Methods

### Patients

This study analyzed SEER data (1973–2013), the open-access cancer registry of the National Cancer Institute (NCI) in the United States. The authors conducted the present analysis after an approval of the “Research Data Agreement” from the SEER. Since the entire raw data were recorded as de-identified, informed consent from the subjects were not required. All of the process has never been involved in identifying personal information of the database. The data extraction and analysis were performed in accordance with relevant guidelines that were published by the SEER^16^.

To extract patient data, we used SEER*Stat software (version 8.3.2; National Institutes of Health, Bethesda, MD)^16^. The raw data included multifarious patients and tumor-related records, including variables of demographics, clinicopathologic information, and survival outcomes. We identified rectal cancer cases based on categories of “C19.9-Rectosigmoid junction” and “C20.9-Rectum, NOS” from the “Primary Site labeled” variable. The histology of adenocarcinomas was identified using the International Classification of Diseases with the malignant behavior code of “/3”. The eligibility criteria included: (1) age >18 years, (2) year of diagnosis from 1998 to 2013, (3) stage II or III based on the 7^th^ American Joint Committee on Cancer (AJCC) staging system, (4) cancer-directed surgery performed, and (5) use of RT after or prior to surgery. Cases without information of primary or lymph node surgery were excluded. Supplementary Fig. 1 represents the diagram of patient selection.

The SEER database summarizes clinical and pathological reports to obtain optimal stage information. We used the variables of AJCC stage and SEER historic or summary stage to exclude cases with initial distant metastasis. Treatment information for each patient was obtainable from the variables “Radiation sequence with surgery,” “Radiation,” and “Reason no cancer-directed surgery.”

### Calculation of propensity scores and adjustment process

In clinical investigations, treatment-related selection bias cannot be ruled out without prospective randomization. A propensity score is the calculated probability of being treated with a certain procedure given a set of baseline covariates^17^. To eliminate potential effects of baseline clinicopathologic variables, propensity score matching method has been widely used in retrospective design^18^. Since the population-based SEER database consists of observational data, we calculated propensity scores and matched patients according to the combined sequence of RT, preoperative vs. postoperative treatment.

For the matching process, prognostic factors evaluated in the initial raw data were applied: age, sex, race, marital status, subsite, histology, extent of primary tumor, lymph node status, tumor grade, types of primary surgery, and lymph node dissection. After calculation of propensity scores with a non-parsimonious logistic regression model, a one-to-one matching process was conducted based on the nearest neighbor method with a caliper 0.2 and without replacement. To assess the balance between the two groups, standardized difference (SD) values for covariates less than 0.1 were considered acceptable after the matching process^19^.

### Statistical analysis

Clinicopathologic characteristics of the pre- and postoperative RT groups were compared with Pearson’s chi-square and Mann-Whitney U tests for categorized and continuous variables, respectively. Overall survival (OS) and disease-specific survival (DSS), defined as the time interval from the diagnosis of cancer to overall and cancer-related death events, respectively, were evaluated as the outcomes of interest. Kaplan-Meier analysis and log-rank tests were used to compare survival differences according to the corresponding covariates, including preoperative vs. postoperative RT. In survival analysis according to nodal status, log odds of lymph nodes were calculated by the formula, “Log [(PLN + 0.5)/(TLN-PLN + 0.5)]”, where “PLN” is the number of positive lymph nodes, and “TLN” is the total number of lymph nodes surgically dissected. Optimal cut-off values of continuous variables, such as age at diagnosis, tumor size, and log odds of lymph nodes, were determined with a maximal chi-square method. For multivariate analysis, the Cox proportional hazards model was applied after the evaluation of proportional hazards assumptions using log-minus-log survival plots of each variable. The potentially associated factors from univariate analyses were included in the Cox-regression analysis of OS and DSS. *P*-values < 0.05 were assessed as statistically significant. IBM SPSS Statistics 22.0 (IBM, Armonk, NY, USA) and R version 3.4.2 (R Foundation for Statistical Computing, Vienna, Austria) were used for all statistical analyses.

## Results

### Study population before propensity score matching

According to the aforementioned eligibility criteria, a total of 28,320 patients were identified. Patient, tumor, and treatment-related characteristics are summarized and compared according to preoperative and postoperative RT methods (Table 1). The median age was 61 (range, 19−99), and 62% of the patients were male. With a predominance of Caucasian patients (82%), 62% were married. Tumor location at the rectosigmoid portion was reported in 5,270 (19%) patients. Not otherwise specified adenocarcinoma histology was diagnosed in 22,875 (81%) patients, and the proportion of tumor grade II (71%) was highest. Approximately 10%, 81%, and 9% of the patients were diagnosed with tumor extension into the submucosa to muscularis propria, pericolorectal tissues, and adjacent organs or structures, respectively, with a median pathologic tumor size of 4.0 cm (range, 0.0−85.0). Stage III with positive lymph node status was observed in 15,523 (55%) patients. Regarding types of primary surgery, 20,570 (73%), 7,117 (25%), and 633 (2%) patients underwent sphincter-preserving, abdominoperineal resection, and pelvic exenteration procedures, respectively. Lymph node dissection was performed in 95% of the patients. The median number of excised and positive lymph nodes was 12 (range, 0−90) and 0 (range, 0−75), respectively.

**Table 1:** Patient, tumor, and treatment-related characteristics between preoperative and postoperative radiotherapy groups before propensity score matching.

Characteristics	Number of patients (%)			*P*
	Total (n = 28320)	Preoperative (n = 17180)	Postoperative (n = 11140)	
Age (years)				
Median (range)	61 (19−99)	60 (19−99)	62 (19−99)	<0.001
Sex				
Male	17471 (62)	10840 (63)	6631 (60)	<0.001
Female	10849 (38)	6340 (37)	4509 (40)	
Race				
Caucasian	23180 (82)	14021 (82)	9159 (82)	0.172
Non-Caucasian	5080 (18)	3117 (18)	1963 (18)	
Unknown	60 (0)	42 (0)	18 (0)	
Marital status				
Married	17543 (62)	10523 (61)	7020 (63)	0.011
Not married	9935 (35)	6134 (36)	3801 (34)	
Unknown	842 (3)	523 (3)	319 (3)	
Site				
Rectum, NOS	23050 (81)	15527 (90)	7523 (68)	<0.001
Rectosigmoid	5270 (19)	1653 (9)	3617 (32)	
Adenocarcinoma histology				
Specified types	5445 (19)	3246 (19)	2199 (20)	0.078
NOS	22875 (81)	13934 (81)	8941 (80)	
Tumor grade				
I	1661 (6)	1028 (6)	633 (6)	<0.001
II	20024 (71)	11841 (69)	8183 (73)	
III	4159 (15)	2261 (13)	1898 (17)	
IV	312 (1)	187 (1)	125 (1)	
Unknown	2164 (7)	1863 (11)	301 (3)	
Extent of primary tumor				
Submucosa to muscularis propria	2729 (10)	1220 (7)	1509 (14)	<0.001
Pericolorectal tissues	23026 (81)	14422 (84)	8604 (77)	
Adjacent organs or structures	2565 (9)	1538 (9)	1027 (9)	
Pathologic tumor size (cm)				
Median (range)	4.0 (0.0−85.0)	4.0 (0.0−55.0)	4.5 (0.0−85.0)	<0.001
Stage				
II	12797 (45)	8171 (48)	4626 (42)	<0.001
III	15523 (55)	9009 (52)	6514 (58)	
Surgical treatment				
Sphincter-preserving surgery	20570 (73)	11744 (68)	8826 (79)	<0.001
Abdominoperineal resection	7117 (25)	4992 (29)	2125 (19)	
Pelvic exenteration	633 (2)	444 (3)	189 (2)	
Lymph node dissection				
Yes	27046 (95)	16153 (94)	10893 (98)	<0.001
No	1274 (5)	1027 (6)	247 (2)	
Number of excised lymph nodes				
Median (range)	12 (0−90)	12 (0−90)	13 (0−90)	<0.001
Number of positive lymph nodes				
Median (range)	0 (0−75)	0 (0−39)	1 (0−75)	<0.001

NOS: not otherwise specified.

### Propensity score matching

Table 2 represents the propensity-matched model of preoperative vs. postoperative RT groups. The matching process identified a total of 18,400 patients who underwent preoperative (n = 9,200) and postoperative (n = 9,200) RT. Given that the overall SD value decreased from 0.628 to 0.061, the distribution of baseline covariates was well-balanced after propensity score matching. For each of the variables, all SD values were less than 0.1 and considered acceptable.

**Table 2:** Distribution of baseline variables before and after propensity score matching.

Characteristics	Before propensity score matching		*Standardized difference*	After propensity score matching		*Standardized difference*
	Preoperative (n = 17180)	Postoperative (n = 11140)		Preoperative (n = 9200)	Postoperative (n = 9200)	
Age (years)						
Mean ± SD	60.1 ± 12.5	61.8 ± 12.2	0.143	61.5 ± 12.3	62.0 ± 12.4	0.042
Sex						
Male	10840 (63)	6631 (60)	0.073	5545 (60)	5453 (59)	0.020
Female	6340 (37)	4509 (40)		3655 (40)	3747 (41)	
Race						
Caucasian	14021 (82)	9159 (82)	−0.018	7608 (83)	7482 (81)	0.034
Non-Caucasian	3117 (18)	1963 (18)		1570 (17)	1701 (19)	
Unknown	42 (0)	18 (0)		22 (0)	17 (0)	
Marital status						
Married	10523 (61)	7020 (63)	−0.036	5717 (62)	5820 (63)	−0.017
Not married	6134 (36)	3801 (34)		3242 (35)	3121 (34)	
Unknown	523 (3)	319 (3)		241 (3)	259 (3)	
Site						
Rectum, NOS	15527 (90)	7523 (68)	0.488	7576 (82)	7510 (82)	0.015
Rectosigmoid	1653 (9)	3617 (32)		1624 (18)	1690 (18)	
Adenocarcinoma histology						
Specified types	3246 (19)	2199 (20)	−0.021	1818 (20)	1973 (21)	−0.042
NOS	13934 (81)	8941 (80)		7382 (80)	7227 (79)	
Tumor grade						
I	1028 (6)	633 (6)	−0.295	677 (7)	569 (6)	−0.041
II	11841 (69)	8183 (73)		6767 (74)	6668 (73)	
III	2261 (13)	1898 (17)		1086 (12)	1563 (17)	
IV	187 (1)	125 (1)		85 (1)	115 (1)	
Unknown	1863 (11)	301 (3)		585 (6)	285 (3)	
Extent of primary tumor						
Submucosa to muscularis propria	1220 (7)	1509 (14)	−0.130	877 (9)	1401 (15)	−0.078
Pericolorectal tissues	14422 (84)	8604 (77)		7688 (84)	6981 (76)	
Adjacent organs or structures	1538 (9)	1027 (9)		635 (7)	818 (9)	
Stage						
II	8171 (48)	4626 (42)	0.122	4013 (44)	3749 (41)	0.058
III	9009 (52)	6514 (58)		5187 (56)	5451 (59)	
Surgical treatment						
Sphincter-preserving surgery	11744 (68)	8826 (79)	−0.258	6852 (74)	6980 (76)	−0.027
Abdominoperineal resection	4992 (29)	2125 (19)		2185 (24)	2044 (22)	
Pelvic exenteration	444 (3)	189 (2)		163 (2)	176 (2)	
Lymph node dissection						
Yes	16153 (94)	10893 (98)	0.255	266 (3)	247 (3)	0.014
No	1027 (6)	247 (2)		8934 (97)	8953 (97)	

SD: standard deviation; NOS: not otherwise specified.

### Kaplan-Meier survival outcomes

**Figure 1:**
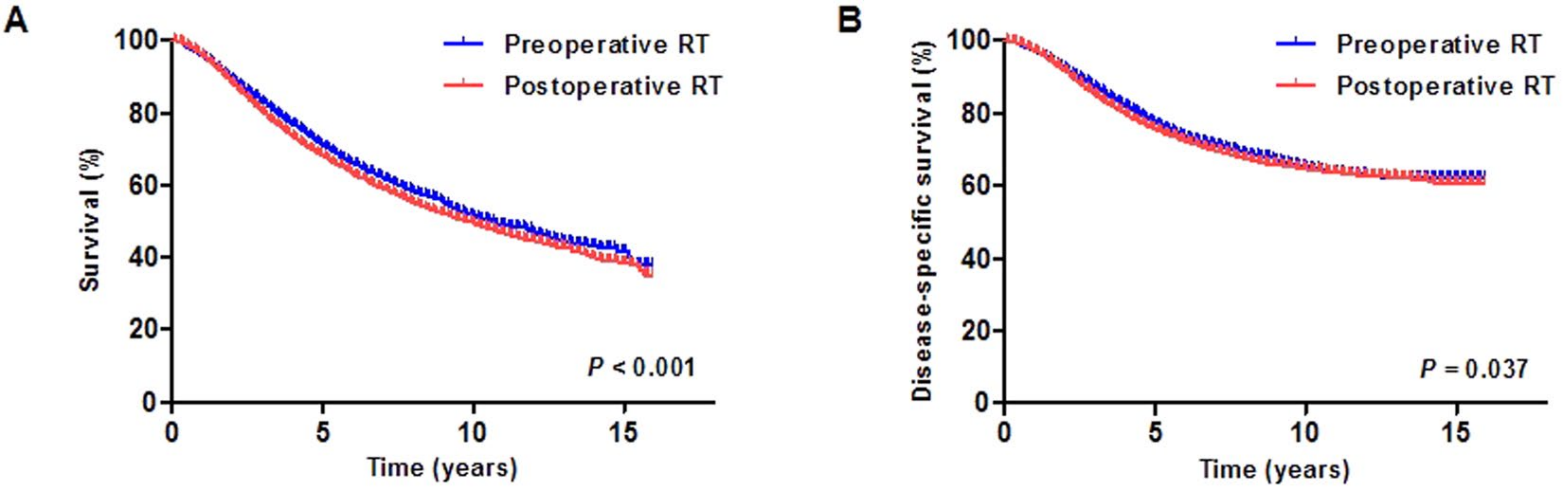
Survival outcomes of stage II-III rectal cancer patients who underwent preoperative and postoperative RT. (**A**) Overall survival and (**B**) disease-specific survival after propensity score matching. RT: radiotherapy.

In the matched population (median follow-up: 4.3 years), the 10-year and 14-year OS rates of preoperative vs. postoperative RT groups were 51.6% vs. 49.8% and 43.1% vs. 40.0%, respectively (*P* < 0.001) (Fig. 1A). The DSS rates at 10 and 14 years were 65.4% vs. 64.8% and 62.0% vs. 61.7%, respectively (*P* = 0.037) (Fig. 1B). For survival analysis, OS and DSS outcomes were compared according to age (≤67 and >67 years), sex (male and female), race (Caucasian and non-Caucasian), marital status (married and not married), site (rectum, not otherwise specified [NOS] and rectosigmoid), types of adenocarcinoma histology (specified and NOS), tumor grade (I, II, and III−IV), extent of primary tumor (submucosa to muscularis propria, pericolorectal tissues, and adjacent organs or structures), pathologic tumor size (<6.0 and ≥6.0 cm), log odds of lymph nodes (≤−0.49 and >−0.49), surgical treatment (sphincter-preserving, abdominoperineal resection, and pelvic exenteration), and combined RT method (preoperative and postoperative). Optimal cutoffs for the above continuous variables were determined by a maximal chi-square method. In univariate analysis of OS, age (*P* < 0.001), sex (*P* < 0.001), marital status (*P* < 0.001), site (*P* = 0.017), histology (*P* < 0.001), tumor grade (*P* < 0.001), extent of primary tumor (*P* < 0.001), pathologic tumor size (*P* < 0.001), log odds of lymph nodes (*P* < 0.001), surgical treatment (*P* < 0.001), and combined sequence of RT (*P* < 0.001) were significant factors. For DSS, the same statistically significant relationships were also observed for most of the above prognostic factors (*P* = 0.037 for combined sequence of RT and *P* < 0.001 for others), except for sex (*P* = 0.402) and site (*P* = 0.513) (Table 3).

**Table 3:** Univariate analysis of the matched cohort.

Variables	N (%)	Overall survival		Disease-specific survival	
		10-year rate (%)	*P*	10-year rate (%)	*P*
Age (years)^*^					
≤67	12170 (66)	60.4	<0.001	68.8	<0.001
>67	6230 (34)	33.5		57.2	
Sex					
Male	10998 (60)	49.0	<0.001	65.0	0.402
Female	7402 (40)	53.4		65.6	
Race					
Caucasian	15090 (82)	50.7	0.946	65.4	0.158
Non-Caucasian	3271 (18)	50.8		64.2	
Marital status					
Married	11537 (63)	54.4	<0.001	67.9	<0.001
Not married	6363 (35)	43.4		60.0	
Site					
Rectum, NOS	15086 (82)	49.9	0.017	65.0	0.513
Rectosigmoid	3314 (18)	54.4		66.5	
Adenocarcinoma histology					
Specified types	3791 (21)	47.8	<0.001	61.2	<0.001
NOS	14609 (79)	51.7		66.3	
Tumor grade					
I	1246 (7)	53.6	<0.001	68.1	<0.001
II	13435 (73)	51.7		66.5	
III−IV	2849 (15)	43.8		57.6	
Extent of primary tumor					
Submucosa to muscularis propria	2278 (12)	63.5	<0.001	75.8	<0.001
Pericolorectal tissues	14669 (80)	50.3		65.3	
Adjacent organs or structures	1453 (8)	36.3		48.4	
Pathologic tumor size^*^					
<6.0 cm	11671 (63)	52.8	<0.001	67.3	<0.001
≥6.0 cm	3829 (21)	44.5		59.9	
Log odds of lymph nodes^*^					
≤−0.49	13334 (73)	56.2	<0.001	71.8	<0.001
>−0.49	4346 (24)	37.2		48.6	
Surgical treatment					
Sphincter-preserving surgery	13832 (75)	53.7	<0.001	67.8	<0.001
Abdominoperineal resection	4229 (23)	42.2		57.7	
Pelvic exenteration	339 (2)	45.1		56.4	
Combined RT sequence					
Preoperative	9200 (50)	51.6	<0.001	65.4	0.037
Postoperative	9200 (50)	49.8		64.8	

^*^Optimal cut-offs were determined with a maximal chi-square method.

NOS: not otherwise specified; RT: radiotherapy.

### Multivariate comparison analysis of preoperative vs. postoperative RT

Table 4 shows the Cox-regression analysis results. For both OS and DSS, the different combinatory strategies of RT did not result in different survival outcomes (hazard ratio [HR] 1.04 and 95% confidence interval [CI] 0.98−1.10 for OS; HR 0.97 and 95% CI 0.90−1.05 for DSS). In terms of OS, age >67 years (*P* < 0.001), not married (*P* < 0.001), tumor grade III−IV (*P* < 0.001), primary tumor extent of pericolorectal tissues and adjacent organs or structures (*P* < 0.001 for both), pathologic tumor size ≥6.0 cm (*P* < 0.001), log odds of lymph nodes > −0.49 (*P* < 0.001), and abdominoperineal resection (*P* < 0.001) induced worse outcomes, and female (*P* < 0.001) and NOS histology (*P* < 0.001) were associated with favorable survival. The prognostic associations of significant variables in OS were the same in DSS (*P* < 0.001 for all comparisons), except for sex.

**Table 4:** Prognostic factors associated with survival in Cox-regression analysis.

Variables	Overall survival		*P*	Disease-specific survival		*P*
	HR	95% CI		HR	95% CI	
Age (years)^*^						
≤67	Ref			Ref		
>67	2.20	2.08−2.33	<0.001	1.63	1.51−1.75	<0.001
Sex						
Male	Ref					
Female	0.81	0.76−0.86	<0.001			
Marital status						
Married	Ref			Ref		
Not married	1.33	1.26−1.42	<0.001	1.28	1.19−1.38	<0.001
Site						
Rectum, NOS	Ref					
Rectosigmoid	0.96	0.89−1.04	0.312			
Adenocarcinoma histology						
Specified types	Ref			Ref		
NOS	0.87	0.81−0.94	<0.001	0.82	0.75−0.90	<0.001
Tumor grade						
I	Ref			Ref		
II	0.99	0.88−1.11	0.859	1.05	0.90−1.23	0.517
III−IV	1.28	1.12−1.45	<0.001	1.40	1.18−1.67	<0.001
Extent of primary tumor						
Submucosa to muscularis propria	Ref			Ref		
Pericolorectal tissues	1.48	1.34−1.63	<0.001	1.65	1.44−1.89	<0.001
Adjacent organs or structures	2.24	1.96−2.55	<0.001	2.81	2.37−3.33	<0.001
Pathologic tumor size^*^						
<6.0 cm	Ref			Ref		
≥6.0 cm	1.24	1.16−1.32	<0.001	1.25	1.15−1.36	<0.001
Log odds of lymph nodes^*^						
≤−0.49	Ref			Ref		
>−0.49	1.90	1.79−2.02	<0.001	2.32	2.15−2.50	<0.001
Surgical treatment						
Sphincter-preserving surgery	Ref			Ref		
Abdominoperineal resection	1.28	1.20−1.36	<0.001	1.35	1.24−1.47	<0.001
Pelvic exenteration	1.14	0.93−1.39	0.209	1.13	0.88−1.45	0.336
Combined RT sequence						
Preoperative	Ref			Ref		
Postoperative	1.04	0.98−1.10	0.238	0.97	0.90−1.05	0.496

*Optimal cut-offs were determined with a maximal chi-square method.

HR: hazard ratio; CI: confidence interval; Ref: reference; NOS: not otherwise specified; RT: radiotherapy.

### Hazard rate of disease-specific mortality risks

**Figure 2:**
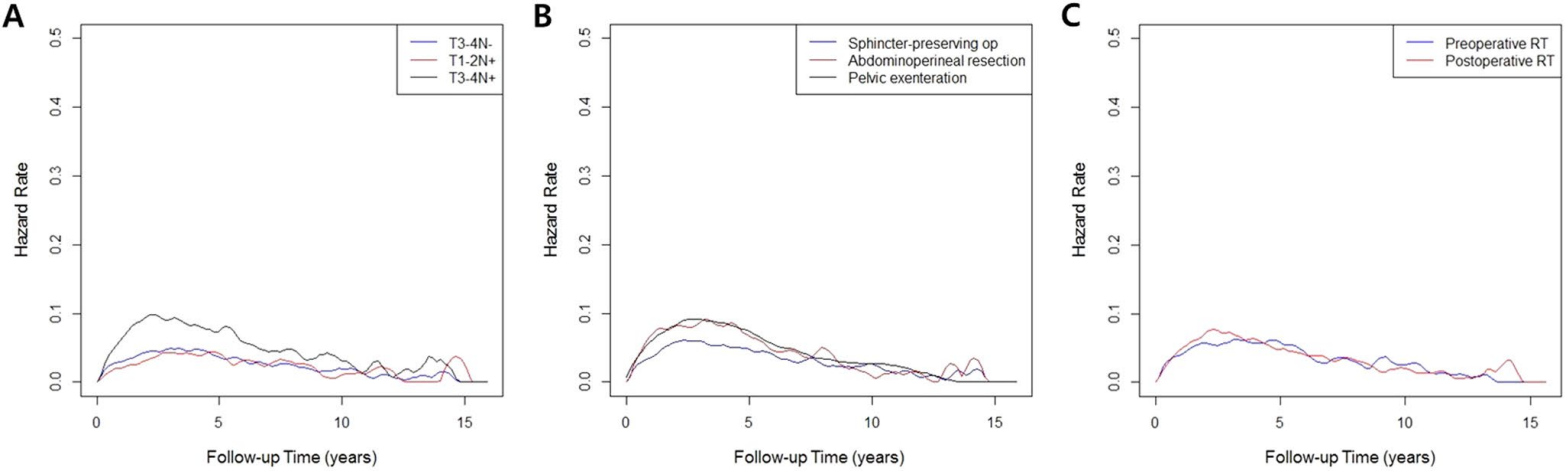
Hazard rate functions of cancer-specific mortality according to (**A**) stage of primary tumor and lymph nodes, (**B**) types of primary surgery, and (**C**) combined sequence of RT. op: operation; RT: radiotherapy.

Figure 2 represents baseline hazard rate function plots of disease-specific mortality according to tumor stage, surgical treatment, and combined RT sequence. The highest and maximal risk increment occurred within 5 years of follow-up, and late risk peaks even after 10 years were observed irrespective of the subgroups. When the patients were stratified according to extent of primary tumor and node status, such as T3-4N−, T1-2N+, and T3-4N+, the overall level of mortality risks was highest with T3-4N+ tumors. Although the overall mortality risk after sphincter-preserving surgery was lower than following the other surgeries, the favorable subgroup also showed late risk peaks around 13−14 years. Overall risk levels were comparable between the pre- and postoperative RT groups along the long-term follow-up period.

## Discussion

We evaluated long-term survival outcomes of stage II-III rectal cancer patients who underwent preoperative or postoperative RT. After propensity score matching, use of RT prior to radical surgery was associated with improved OS and DSS in univariate Kaplan-Meier analysis. However, the favorable prognostic impacts were not maintained after adjusting for other related clinicopathologic covariates. There were no definite differences in the time-course patterns of cancer-specific mortality between the two treatment groups. The present study is the first comparative survival analysis of preoperative and postoperative RT in locally advanced rectal cancer, using long-term population-based data.

Among the three historical phase III trials comparing pre- and postoperative RT^9–11^, the German CAO/ARO/AIO-94 trial was the largest one, suggesting clinical benefits of the preoperative approach in local tumor control (*P* = 0.006), down-staging effect (*P* < 0.001), conversion rate of sphincter preservation (*P* = 0.004), and severe acute and late toxicity (*P* = 0.001 and 0.01, respectively)^9^. However, long-term analysis of the same data found no survival difference between the two RT strategies (59.9% vs. 59.6% for preoperative vs. postoperative, *P* = 0.85)^13^. Although the NSABP R-03 study suggested a trend towards improved survival with preoperative RT (74.5% vs. 65.6%, *P* = 0.065), the results have not been considered decisive due to the poor accrual of patients^10^. In another Korean trial, significant differences did not exist in disease-free survival (*P* = 0.866), local control (*P* = 0.393), and OS (*P* = 0.620), but preoperative RT resulted in higher rates of sphincter preservation for low-lying tumors (*P* = 0.008)^11^. Although the German trial contributed most significantly to the current clinical guidelines in favor of use of preoperative RT^3^, the beneficial effect in local tumor control was mainly confined to the intention-to-treat analysis^9^. Additionally, whether enhanced oncologic outcomes with a preoperative approach induce long-term survival benefits remains unclear. Therefore, a comparative prognostic assessment of the two different RT strategies is needed in contemporary clinical practices.

The SEER database contains large-scale data on a variety of malignancies^15^. Given that the registry includes accurate treatment information, we used the data to compare the two RT approaches in stage II-III rectal cancer. The large patient population is a strength of our study, and survival outcomes after longer durations of follow-up can be informative. Nevertheless, under the retrospective design, results of univariate analysis cannot be conclusive due to the existence of various confounding factors and related selection bias. In this study, the preoperative RT strategy seemed to be associated with lower mortality risks in the univariate analysis, but did not significantly affect long-term prognosis after adjusting for other related factors. The absence of significant survival differences between the two groups is consistent with conclusions of prior investigations^11–13,20^.

Current clinical guidelines endorse preoperative RT as the preferred option^3^. Irradiation before surgical resection sterilizes gross and microscopic tumor cells under the better condition of tumor oxygenation, suggesting potential benefits in preventing further tumor spread within the locoregional RT field^5,9^. In clinics, main reasons for the recommendation have been derived from some favorable oncologic outcomes: down-staging effect, increased likelihood of sphincter preservation, and relatively better treatment compliance expected^6,7,9–11^. In this study, the unadjusted results indicating better survival with preoperative RT might be attributable to favorable factors related to the use of RT prior to surgery. However, adjusting for a variety of baseline characteristics, including demographic data, detailed pathologic features, extent of tumor and nodal status, and types of surgical treatment, we conclude that the selection preoperative or postoperative RT did not affect overall or cancer-related death events. Here, from long-term analysis, we suggest that the RT strategy in each case needs to be determined at the discretion of the physician or surgeon with informed consent of patient, considering the need of sphincter preservation and compliance of surgery or RT at the institution.

To estimate time-course changes of cancer-specific mortality risks, baseline hazard rate functions were plotted and analyzed. When individual hazard rate curves were drawn according to tumor extent and combined RT approaches, most of the short-term risk peaks were maximized within 5 years of follow-up, and a sustained long-term risk increment even after 10 years was noticeable. The overall risk patterns evaluated in a time-dependent manner were comparable between the two RT groups, whereas a late risk peak was revealed in the adjuvant RT group after approximately 14 years. Our results highlight the importance of continued surveillance with a longer follow-up duration, and further suggest the need for a systematic strategy to prevent the potential of late failure. Further multi-institutional investigations are needed to elucidate the long-term failure patterns in locally advanced rectal cancer^21,22^.

To date, several prior investigations have used the SEER registry to analyze rectal cancer patients and the combined use of RT^23,24^. Peng *et al*. compared T3N0 rectal cancer patients who underwent surgery alone, preoperative RT followed by surgery, and surgery plus postoperative RT^23^. They found that use of postoperative RT was associated with improved 10-year DSS rates compared with surgery alone (76.1% vs. 66.1%, *P* < 0.001), whereas there was no survival benefit of preoperative RT (*P* = 0.127). However, the study did not conduct any calculation to reduce treatment-related selection bias. Another SEER study evaluated the effectiveness of preoperative RT compared with surgery alone, in stage II-III rectal cancer^24^. The study performed propensity score matching, and the use of preoperative RT led to improvement in DSS even after adjusting for other clinicopathologic factors (HR 0.741, 95% CI 0.646−0.811). Based on patient age (>50 and ≤50 years), the benefits of RT were confined to older patients (*P* = 0.006 and <0.001 for stage II and III, respectively). Although the potential for differential prognostic effects in different age groups was a novel finding, such exploratory subgroup analysis cannot be conclusive using a retrospective design. In addition, the comparison of preoperative RT with surgery alone is less clinically relevant, in that neoadjuvant or adjuvant RT has been generalized in the contemporary treatment of locally advanced rectal cancer.

Staging discrepancies between clinical and pathological tumor status exist in analyzing patients who underwent preoperative RT. In the era of neoadjuvant treatment for rectal cancer, post-RT tumor regression grade or neoadjuvant rectal (NAR) score is considered as a surrogate marker for prognosis^25,26^. The SEER summarizes clinical and pathological stage information for some specific subsets of patients who underwent any preoperative therapy (RT, chemotherapy, hormone therapy or immunotherapy). Due to varying degrees of individual treatment response, the largest or greatest extent of disease was coded regarding tumor status prior to and after preoperative treatment^27,28^. Although we applied demographic and tumor-related variables as possible in the matching process, potential selection bias from the unknown information of pathological response was inevitable.

Tumor location from the anal verge is one of the important factors to decide treatment strategies. In this study, however, the type of surgery was adjusted in the propensity score matching and Cox-regression analysis, which partially enabled the consideration of low-lying tumors. Besides, variations in clinical practices due to different institutional policies or a clinician’s discretion can also influence the selection of either preoperative or postoperative RT. Patients with more risk features at initial diagnosis might have higher tendency to undergo preoperative treatment, which was not adjusted in the present analysis. The absence of chemotherapy information and RT regimens (short-course or long-course), as per the policy of SEER, was another weakness^29^. Although pelvic recurrence is an important outcome of interest in other clinical investigations of rectal cancer, failure events or recurrence-free survival data were not available in the SEER database. Other individual health data, such as underlying comorbid illness, performance status, toxicity profiles, and any reasons that the patient could not undergo preoperative treatment first, and vice versa, were not obtainable. In fact, the incompleteness of data is an inevitable limitation of the population-based studies. Nevertheless, our study has its value in that the results were based on large-scale long-term survival data under the contemporary treatment techniques.

We compared the impacts of pre- and postoperative RT on patient survival in stage II-III rectal cancer using the large-scale SEER database. The different sequence of RT relative to surgery did not independently affect long-term OS and DSS, and the time-course patterns of mortality risks were comparable between the two treatment groups. Along with a sustained long-term risk increment in patients overall, the occurrence of a late risk peak near the end of follow-up suggests the need for optimal systemic management to prevent late failure events. Our population-based results are supportive of equivalent survival outcomes of the two combined approaches in locally advanced rectal cancer, which provides additional insights into the long-term prognostic implications of RT strategies. Further studies, such as ongoing trials of total neoadjuvant therapy^30^, are needed to improve therapeutic efficacy of the current standard treatment.

## Electronic supplementary material

Supplementary Figure 1
