# Supplementary material for: Comparative survival analysis of preoperative and postoperative radiotherapy in stage II-III rectal cancer on the basis of long-term population data
Source: Sci Rep. 2018 Nov 21;8:17153. doi: 10.1038/s41598-018-35493-2 (PMC6249278; doi:10.1038/s41598-018-35493-2)
Supplement: Supplementary file 1 — Supplementary Figure 1 [file 41598_2018_35493_MOESM1_ESM.docx]

**Comparative survival analysis of preoperative and postoperative radiotherapy in stage II-III rectal cancer on the basis of long-term population data**

Yu Jin Lim, Youngkyong Kim, and Moonkyoo Kong^*^

Department of Radiation Oncology, Kyung Hee University Medical Center, Kyung Hee University School of Medicine, Seoul, Republic of Korea

***Correspondence:** Moonkyoo Kong

Department of Radiation Oncology, Kyung Hee University Medical Center, Kyung Hee University School of Medicine, 23 Kyungheedae-ro, Dongdaemoon-gu, Seoul, 02447

Telephone: +82-2-958-8661

Fax: +82-2-958-9469

E-mail: [kongmoonkyoo@khu.ac.kr](mailto:kongmoonkyoo@khu.ac.kr)


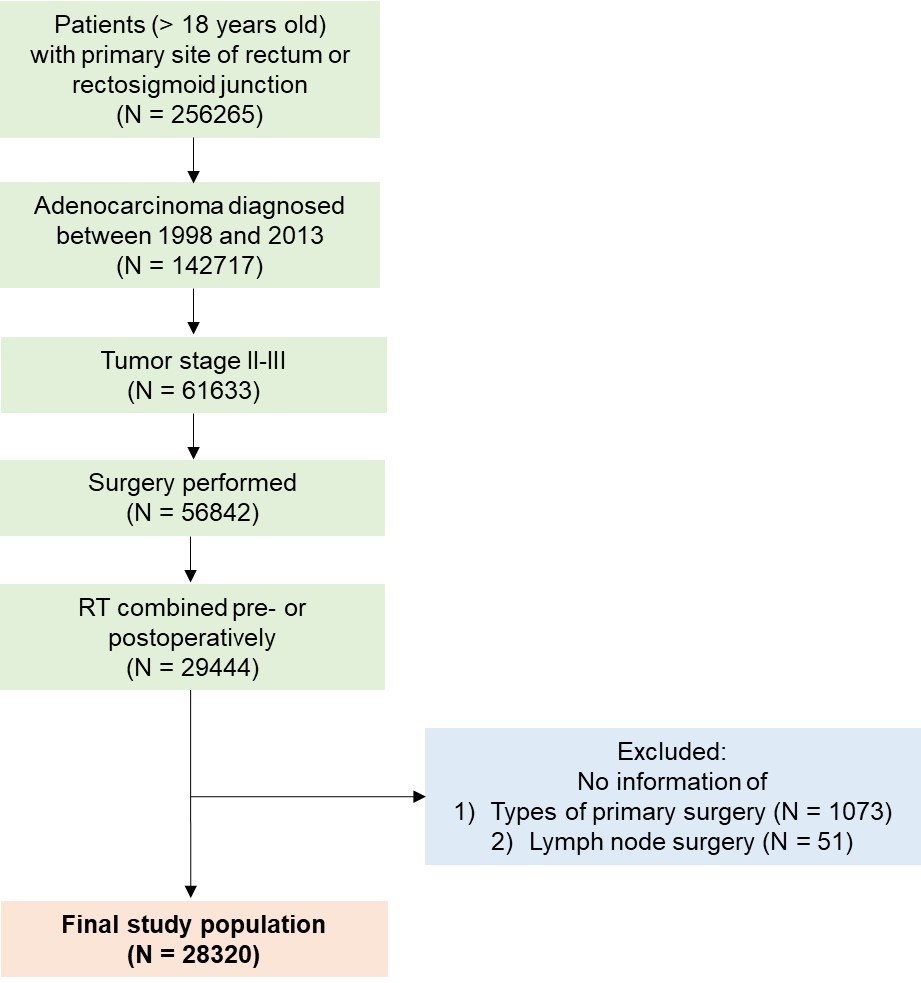


**Supplementary Figure 1:** Flow diagram of patient selection. RT: radiotherapy.
